# Technical Challenges in Studying Infectious Disease-Associated Pulmonary Hypertension in Low- and Middle-Income Countries with Limited Resources

**DOI:** 10.3390/idr17050109

**Published:** 2025-09-10

**Authors:** Jennifer van Heerden, Irina Mbanze, Elizabeth Louw, Olukayode Aremu, Anastase Dzudie, Ana Mocumbi, Threnesan Naidoo, Brian Allwood, Friedrich Thienemann

**Affiliations:** 1 Department of Paediatrics, University of Oxford, Oxford OX1 2JD, UK; 2 Division of Cardiology, Department of Medicine, Maputo Central Hospital, Maputo 1164, Mozambique; 3 Cape Heart Institute, Department of Medicine, University of Cape Town, Cape Town 7925, South Africa; 4 Division of Pulmonology, Department of Medicine, University of Stellenbosch, Cape Town 7505, South Africa; 5 Department of Internal Medicine and Cardiology, Douala General Hospital, Douala 00556, Cameroon; 6 Faculty of Medicine, Universidade Eduardo Mondlane, Maputo 1102, Mozambique; 7 Department of Forensic & Legal Medicine, Faculty of Medicine & Health Sciences, Walter Sisulu University, Mthatha 5099, South Africa; 8 Africa Health Research Institute, Durban 4001, South Africa; 9 General Medicine & Global Health, Department of Medicine, University of Zurich, 8091 Zurich, Switzerland

**Keywords:** pulmonary hypertension, infectious disease, low- and middle-income countries, tuberculosis, HIV, schistosomiasis, rheumatic heart disease

## Abstract

Background: Pulmonary hypertension (PH) disproportionately affects those residing in low- and middle-income countries (LMICs). Given that these countries also have a high prevalence of infectious diseases, many cases of PH are either directly or indirectly related to infectious etiologies. Despite this correlation, the precise burden of infectious disease-associated PH is largely underappreciated due to a lack of diagnostic resources, a shortage of clinical expertise to carry out right heart catheterization and poor access to healthcare facilities in many low- and middle-income settings. Methods: In this narrative review, we highlight the significant burden of infectious disease-associated PH in LMICs, outline the technical challenges faced by LMICs when diagnosing PH, and propose possible solutions for diagnosing PH in resource-constrained settings. Conclusions: Low-cost and sustainable solutions for infectious disease-associated PH in LMICs should be prioritized. Meaningful solutions require collaborative efforts and capacity building in LMICs.

## 1. The Burden of Infectious Disease-Associated Pulmonary Hypertension in Low- and Middle-Income Countries

Pulmonary hypertension (PH) is a debilitating, progressive, and often life-shortening condition which significantly impacts the quality of life and functioning of those affected by the disease [1]. PH disproportionately affects low- and middle-income countries (LMICs)—defined by the World Bank as a gross national income per capita of less than approximately USD 14,000—with an estimated 80% of people living with PH residing in LMICs [2]. Moreover, PH in these settings typically affects a younger age group (<65 years old) compared to high-income settings, resulting in PH carrying a high socio- and economic impact in these countries [2]. Despite this high burden, the precise prevalence of PH in LMICs remains unknown and is considered to be largely underestimated due to the inaccessibility of accepted diagnostic techniques in resource-constrained settings—defined as any setting where there are significant limitations on the availability of necessary health resources, including personnel, equipment, and/or facilities. In this narrative review, we outline the technical challenges to the detection of PH relating to infectious diseases etiologies in LMICs with limited resources; and propose possible solutions for the approach to infectious disease-associated PH in these settings.

**Figure 1:**
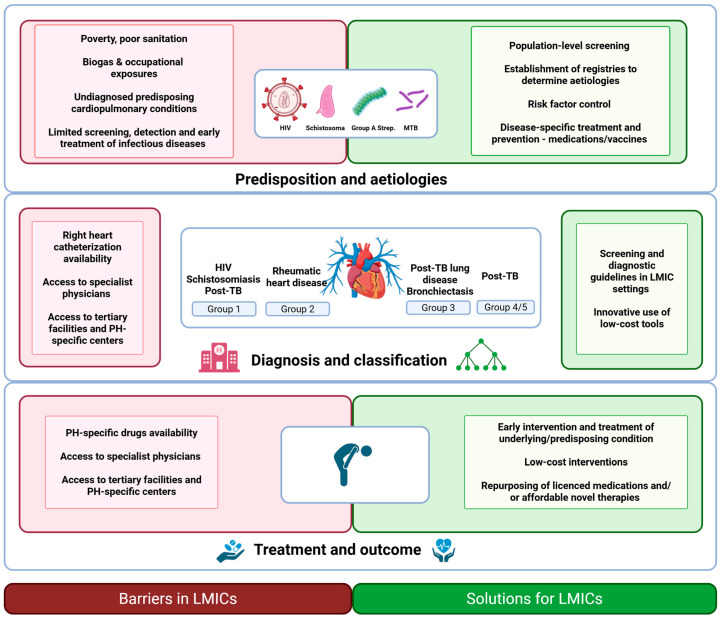
An overview of the barriers and potential solutions in the diagnosis and management of infectious disease-associated pulmonary hypertension (PH) in low- and middle-income countries (LMICs). Created with BioRender.com (BioRender, Toronto, Canada, accessed on 1 March 2025). Abbreviations: HIV, Human Immunodeficiency Virus; LMIC, low- and middle-income countries; MTB, *Mycobacterium tuberculosis*; PH, pulmonary hypertension; TB, tuberculosis.

Although left heart disease is considered the most common cause of PH globally, the epidemiology and etiologies of PH in LMICs are diverse and include important predisposing risk factors and conditions which are not mirrored in higher-income settings [2,3]. Most notably, infectious etiologies contribute significantly to the high prevalence of PH in LMICs, yet these are often underappreciated and undertreated due to multiple barriers in the pathway of care (Figure 1). The burden of infectious disease-associated PH is primarily driven by *Streptococcal*-associated acute rheumatic fever and its consequence, rheumatic heart disease (RHD) (prevalence estimated at 12.9% (95%CI 11.8–14.0) of 3750 patients with RHD in Africa in a systematic review by Bigna et al.), HIV infection (prevalence approximately 10.6% (95%CI 4.3–19.1) of 937 individuals living with HIV and presenting with cardiac complaints), and *Schistosoma* spp. (prevalence of PH in patients with hepatosplenic chronic schistosomiasis, estimated at 8–25%) [4,5]. More recently, the substantial burden of tuberculosis (TB)-associated PH has been highlighted, with a recent systematic review and meta-analysis showing a prevalence of PH of approximately 10% in those with active TB and up to 67% in those with severe post-TB lung disease (PTLD) [6].

Although the above four pathogens—*S. progenes*, HIV, *Schistosoma* ssp. and MTB—will be the focus of this narrative review, other infectious diseases also affect the pulmonary vasculature and may directly or indirectly lead to PH. These agents include viral infections, most notably SARS-CoV-2, fungal infections such as pulmonary paracoccidioidomycosis and *Aspergillus*, and bacterial infections including nontuberculous mycobacteria [3,7,8]. Additionally, repeated bronchopulmonary infections causing bronchiectasis and infection-related myocarditis with the development of heart failure are other potential mechanisms by which infectious etiologies contribute to the overall burden of PH.

## 2. Infectious Disease-Associated Pulmonary Hypertension: Definitions and Classification

### 2.1. Pulmonary Hypertension

According to the most recent European Society of Cardiology and European Respiratory Society (ESC/ERS) guidelines, PH is defined at right heart catheterization (RHC) by a mean pulmonary arterial pressure (mPAP) ≥ 20 mmHg at rest [9]. PH is further subclassified into five groups, according to pathophysiological mechanisms, clinical presentation, hemodynamic characteristics, and therapeutic management [9].

### 2.2. HIV and Schistosomiasis-Associated Pulmonary Hypertension

HIV and schistosomiasis cause pulmonary arterial hypertension (PAH) (i.e., Group 1 PH, PAH-HIV or schistosomiasis-associated PAH respectively), characterized at RHC by pre-capillary PH (mPAP ≥ 20 mmHg; PAWP ≤ 15 mmHg; PVR > 2 WU) [9]. PAH-HIV previously has been considered as a rare complication of HIV, with an estimated 0.5% of people living with HIV being affected by the condition when this condition is diagnosed at RHC [10]. However, limiting prevalence estimates to studies and settings where diagnosis at RHC is available has likely resulted in an underestimation of disease and the prevalence estimate is up to 10.6% when detected at echocardiography [4]. This discrepancy highlights that many cases of PAH-HIV are likely missed, which may have notable implications for at-risk individuals living in lower-income settings.

Schistosomiasis-associated pulmonary arterial hypertension (Sch-PAH) is one of the leading causes of Group 1 PH and is estimated to affect approximately 425,000 people worldwide [11,12]. In particular, in high prevalence areas such as Brazil, Sch-PAH causes approximately 20% to 30% of all PH cases referred to PH centers [11]. Despite this high prevalence, many diagnoses of Sch-PAH are missed due to the condition predominantly occurring in areas where resources are limited. This is particularly applicable to a RHC-dependent diagnosis, which requires invasive testing and is not appropriate for population screening.

### 2.3. Rheumatic Heart Disease-Associated Pulmonary Hypertension

Rheumatic heart disease is a leading cause of Group 2 PH in many LMICs. It is mostly diagnosed in the setting of multivalvular disease and heart failure, and is characterized hemodynamically by post-capillary PH (mPAP ≥ 20 mmHg; PAWP > 15 mmHg), which can be isolated (ipcPH) or combined with pre-capillary PH (cpcPH) [9,13]. PH is a poor prognostic sign in RHD, with PH-RHD being a major cause of morbidity and mortality in LMICs. Unfortunately, RHD-PH is often detected late, and data from the Global Rheumatic Heart Disease Registry showed that of 3343 patients with RHD from 12 African countries, 28.8% already had PH at presentation [13].

### 2.4. TB-Associated Pulmonary Hypertension

TB typically causes Group 3 PH (mPAP ≥ 20 mmHg; PAWP ≤ 15 mmHg; PVR >2 WU) and occurs within the setting of post-TB lung disease (PTLD). However, more rarely, active or previous TB may cause mediastinal fibrosis (i.e., Group 5 PH); may predispose patients to chronic thromboembolic PH (i.e., Group 4 PH) and PH may additionally develop in the context of active TB infection [6,14]. Moreover, in some cases where PH is disproportionate to the severity of the structural and functional pulmonary dysfunction of PTLD, PAH (i.e., Group 1 PH) may develop; the mechanism is unknown, but possibly attributable to pulmonary vascular disease or ongoing low-grade inflammation from previous pulmonary TB [14]. Indeed, in some cases of post-TB PH, plexiform lesions and medial thickening are identified under histopathological examination (Naidoo et al., unpublished). Unravelling the complexities of the multifactorial pathophysiology of post-TB PH requires tissue-based studies in which a comprehensive morphological, cellular, and molecular characterization of the spectrum of vascular alterations can be correlated against relevant clinical, biochemical, and imaging parameters as well as functional assessments. However, research pipelines for the patho-molecular analysis of human lung tissue are very costly and specific animal models for mechanistic studies of PH in the context of PTLD are lacking [15,16].

Although the association between TB and pulmonary vascular disease is now acknowledged; no clear screening algorithms for PH exist in high-TB-prevalence areas. This is particularly important because approximately 155 million people have had a previous episode of TB and probable post-TB PH was found to be present in up to 9% of minimally symptomatic or asymptomatic outpatients at echocardiography [17].

## 3. Diagnostic Importance of Infectious Disease-Associated Pulmonary Hypertension in LMICs

Accurate diagnosis, classification, and etiological investigation of PH require an array of imaging and laboratory modalities, many of which should preferably be carried out in specialist PH centers. Specifically, the ESC/ERS guidelines recommend at least the following in a three-step diagnostic process from initial PH suspicion (clinical examination, blood test for BNP or NT-proBNP, ECG) to PH detection (non-invasive pulmonary and cardiac assessment including echocardiography) and PH confirmation (RHC). Thereafter, a comprehensive evaluation of the etiology is required, including imaging assessments (e.g., computed tomography, lung scintigraphy), functional assessments (e.g., cardiopulmonary exercise testing, pulmonary function tests, and arterial blood gas analysis), and comprehensive laboratory and genetic testing [9].

Given the high cost of many of the investigations required in PH detection and classification, it is unsurprising that the identification, diagnosis, and characterization of PH in limited-resource settings is challenging, which results in high rates of delayed and missed diagnoses. This ultimately results in both an underestimation of the burden of PH and poor patient outcomes. Several additional factors contribute to the diagnostic delay of PH in LMICs, including patients’ lack of access to healthcare and the relatively non-specific nature of PH symptoms, such as shortness of breath and fatigue, which are diagnosed as more commonly occurring conditions.

Regardless of the underlying disease, the development of PH is commonly associated with a poor prognosis and an increased risk of mortality. In LMICs such as those in sub-Saharan Africa, particularly high mortality rates of PH are reported [18]. This most likely reflects the fact that patients in these settings usually present at late stages of the disease (WHO functional class III and IV) [18,19]. This was one of the most notable finding in a large African registry, where at 6-month follow-up, 21% of adults diagnosed with PH with follow-up data had died and mortality was significantly associated with increasing functional impairment [18]. Similarly, in the PROKERALA Registry from Southern India, rehospitalization was more than 60% in the first year after diagnosis, and more than 70% of patients presented with a New York Heart Association functional class of III/IV in a large Argentinian registry [20,21]. Therefore, the first—and the most important—reason for reducing diagnostic delays and promptly diagnosing PH is to improve patient outcomes in low-income settings.

The second reason that the timeous diagnosis of PH is important stems from a global health and research-based perspective. Accurately defining the burden of PH in LMICs, precisely delineating the predisposing conditions, and correctly identifying barriers in the pathway of PH care is critical to lessening the overall impact of this disease. This was exemplified by the Pan African PH Cohort study (PAPUCO), a multinational multicenter registry of PH across Africa, which allowed for the evaluation of the causes, presentation, severity, management, and natural course of PH in Africa [18]. Importantly, due to this study being carried out in low-resource settings, PAPUCO used a modified diagnostic algorithm to diagnose and classify PH, which also took into consideration high-burden conditions in the specific African country where it was being utilized. Other more recent working groups have also identified the lack of both clinical and preclinical data in the field of infection-related pulmonary vascular disease as an important global health concern, with an urgent need to improve collaborative efforts to clearly define the epidemiology and burden of infectious diseases which affect the pulmonary vasculature [3,14]. At present, an acknowledged gap exists in the evidence informing PH care in RHD-PH and post-TB PH; however, accurately defining the burden of disease would highlight the need for funding and support in conducting interventional trials in these populations.

## 4. Right Heart Catheterization and Non-Invasive Alternatives for the Detection and Diagnosis of Pulmonary Hypertension

Accurate diagnosis of PH is critical for effective management. However, the challenges faced in LMICs—namely resource limitations, limited infrastructure, reduced access to specialized care, as well as limited therapeutic options—make the exploration of affordable and non-invasive imaging modalities essential for improving detection rates in these regions.

### 4.1. Right Heart Catheterization

Right heart catheterization (RHC) is accurate in assessing various forms of PH. However, access to this critical tool is often inadequate in LMICs, where healthcare resources are limited. This procedure involves inserting a catheter into the right side of the heart and pulmonary artery to measure pressures and assess cardiac function, and is well-recognized as the gold standard for diagnosing PH. RHC not only provides a definitive measurement of mPAP at rest, which is essential for diagnosing PH, but is needed to measure PAWP and PVR [22,23]. Thus, RHC is important for comprehensive hemodynamic profiling and aids in distinguishing between pre-capillary and post-capillary causes of PH, which is crucial for appropriate treatment strategies [23,24]. However, despite its importance, limited resources, lack of healthcare access, and epidemiological factors are part of the challenges existing in implementing RHC in LMICs. The absence of the necessary equipment and trained personnel in many healthcare facilities in LMICs impairs the ability to perform RHC safely and effectively, leading to a reliance on less accurate methods such as echocardiography, which may not provide sufficient detail for a definitive diagnosis [22,24]. Patients in LMICs often face barriers to accessing specialized care, including long waiting times and geographical limitations such as infrastructural and transportation problems, which in turn could delay diagnosis and treatment, and worsen health outcomes [22].

### 4.2. Echocardiography

Echocardiography is the most widely available and commonly used non-invasive imaging modality for the detection of PH [25]. Echocardiography uses ultrasound waves to create images of the heart and assess its function and structure [26]. Echocardiography is available, portable, cost-effective and can perform real-time and parametric assessments by providing immediate results, allowing for prompt and guided clinical decision-making. However, despite these benefits, echocardiography has a lower sensitivity [81% (95% CI 70–88%)] and specificity [61% (95% CI 53–69%)] in PH diagnosed in patients with underlying lung disease compared to those without [27,28].

By measuring the tricuspid regurgitant velocity (TRV) through Doppler ultrasound, clinicians can estimate the pulmonary artery systolic pressure (PASP) [26]. However, this measurement may not be possible in up to 40% of patients with pulmonary disease [29], and measurements such as right ventricular outflow tract acceleration time (RVOT-AT), which is measurable in more than 90% of patients with lung disease, should be considered as an alternative option in these cases, and has an estimated sensitivity and specificity of 84% [30].

Although echocardiography is operator-dependent and has limited accuracy and ability to differentiate between various diseases in certain patient populations, such as obesity and lung disease [31], it remains the gold standard for initial screening and ongoing monitoring of patients with PH in LMICs [32].

### 4.3. Computed Tomography (CT) Angiography

CT angiography has become a notable tool for the detection of PH. CT angiography has the ability to visualize the pulmonary vasculature and assess certain underlying causes of PH, such as pulmonary embolism, vascular malformations, and chronic thromboembolic pulmonary hypertension (CTEPH) by identifying perfusion defects [33]. Dual-energy CT has shown promising findings in identifying CTEPH with a high sensitivity and specificity, making it a possible non-invasive alternative when RHC is unavailable [31]. The ability to use CT not only for anatomical assessment but also for functional evaluation of the pulmonary vasculature is crucial for comprehensive patient management. Despite these advantages, CT is expensive and often limited due to exposure to ionizing radiation, which poses a significant concern in young and/or high-risk populations and, in PTLD, CTPA, although very specific, has a high false-positive rate for detecting PH (positive predictive value ±40%) [34].

### 4.4. Cardiovascular Magnetic Resonance Imaging

Cardiovascular magnetic resonance imaging (CMR) has emerged as a valuable, non-invasive tool in the clinical detection of PH, aiding accurate diagnosis and classification of PH for effective management. CMR provides a comprehensive evaluation of right ventricular (RV) morphology, function, and volume, being the gold standard for quantifying these parameters, which are critical for understanding the prognosis of PH patients [35,36]. Compared with other imaging modalities, CMR can be utilized in monitoring disease progression and treatment non-invasively [37,38]. Changes in RV function observed via CMR can indicate disease progression and guide therapeutic decisions [39]. Assessment of RV mass and strain have been shown to correlate with long-term outcomes in patients with PH [36]. Assessment of pulmonary hemodynamics and lung perfusion using advanced CMR techniques (not involving the use of contrast agents) contributes to a more comprehensive understanding of the patient’s condition. Jahnke and colleagues (2022) reported a 90% acute procedural success rate in 32 patients prior to interventional treatment of pulmonary vein stenosis at baseline, 1-day and 3-month follow-up [40]. However, despite its usefulness, CMR is limited in LMICs due to financial constraints and lack of skilled personnel, inadequate awareness regarding the benefits of CMR among health providers, and lack of maintenance and management systems.

### 4.5. Biomarker Utilization 

Biomarkers are useful, non-invasive tools and an accurate biomarker would assist in providing information about the diagnosis, disease severity, response to treatment, and prognosis of PH. However, due to the complex and heterogeneous nature of PH, no single biomarker accurately reflects these parameters. N-terminal-pro-brain-type natriuretic peptide (NT-proBNP) is the most widely utilized biomarker in the context of PH, and may correlate with both prognosis and response to treatment in certain PH populations [41]. The search for other, more novel biomarkers is ongoing, with promising advancements in the field [42]. However, in an LMIC-setting, highly specialized biomarkers (such as endothelin-1 and growth differentiation factor-15) are often unavailable due to the limitations of local laboratory assays and, although more affordable and available biomarkers (such as CRP, red cell distribution width, and uric acid concentrations) have been investigated, these lack specificity for PH. Therefore, as the PH community investigates new biomarkers, taking cost and availability into account should be prioritized in order to develop solutions that are accessible to lower-resource settings.

## 5. Resource Limitations and the Challenges in the Diagnosis and Management of Pulmonary Hypertension in LMICs

The diagnosis and management of PH require a holistic and multidisciplinary approach [9]; however, LMICs are not capable of facing the disease burden due to a critical shortage of resources and personnel [43]. Challenges range from a lack of resources to cost of care, scarcity of expertise, unpredictable availability of treatment options, and the extremely rare option of lung transplantation. Additionally, lack of data on PH in LMICs leads to extrapolation of etiology, diagnosis, and management algorithms from high-income countries (HICs) which might not address some of the particularities of LMICs [44].

**Figure 2:**
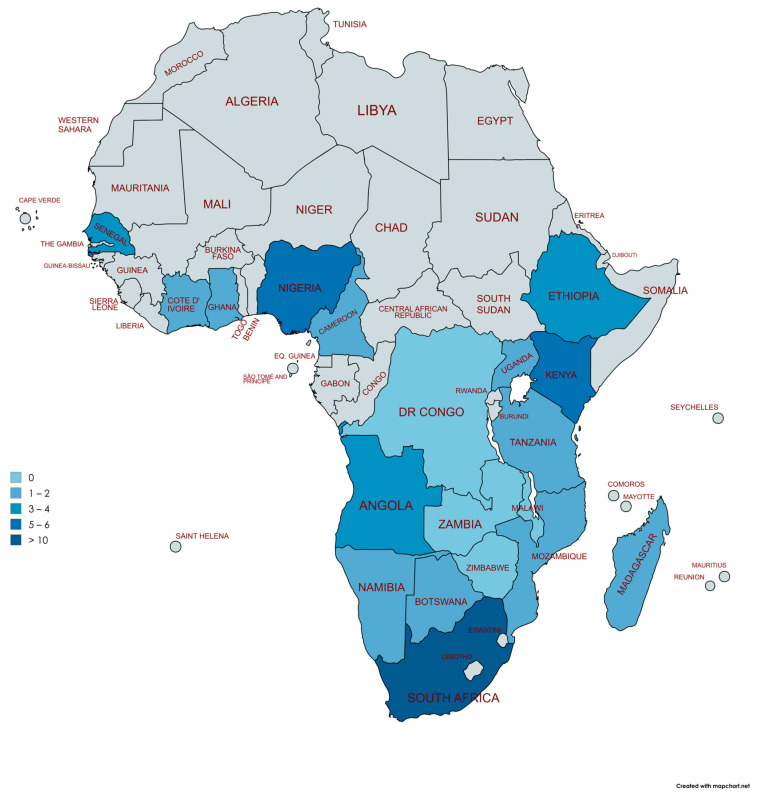
Distribution of cardiac catheterization laboratories in sub-Saharan African countries. Adapted from Dr Graham Cassel, Milpark Hospital, Johannesburg and Stassen et al. [46]. Democratic Republic (DR) of the Congo, Zambia, Zimbabwe, and Malawi (*n* = 0), Botswana, Madagascar, Uganda, Cameroon, and Cote D’Ivoire (*n* = 1), Namibia, Mozambique, Tanzania, Ghana (*n* = 2), Ethiopia (*n* = 3), Angola, and Senegal (*n* = 4), Kenya (*n* = 5), Nigeria (*n* = 6), South Africa (*n* > 10). Created with mapchart.net (MapChart version 6, accessed on 26 April 2025).

Despite the key role of cardiac catheterization laboratory facilities for the diagnosis of PH, there is critical shortage of these facilities in LMICs, particularly in sub-Saharan Africa [43]. The majority of the LMICs fall remarkably below the minimum recommended standard of one cardiac catheterization laboratory per 1,000,000 population (Figure 2) [45]. Uganda is an example, with a population of 42 million inhabitants in 2021 and only one operational catheterization laboratory facility available. A similar situation is found in Cameroon, Botswana, Tanzania, Mozambique, Namibia, and other sub-Saharan African countries. Data from 2017 indicate that South Africa has 62 (~887,096 persons/facility) cardiac catheterization facilities; however, three-quarters of these are contained within the private sector and therefore are unavailable to the majority of South Africans [46]. Interestingly, with the correct knowledge and expertise, RHC does not necessarily require a delegated catheterization laboratory, and can be safely performed in other procedural settings—provided that these facilities have sufficient staff and equipment [47].

Echocardiography remains a valuable tool to diagnose PH, particularly PH associated with left heart disease and congenital heart disease [9]. Although it has become increasingly available in LMICs, access remains insufficient. In the PAPUCO study, Thienemann et al. [18] demonstrated that in the absence of catheterization laboratory facilities—a reality in the majority of the participating countries—echocardiography was pivotal for diagnosis of PH.

Although we have witnessed significant technological advances in HICs, there is a scarcity of non-invasive diagnostic tests for detection and confirmation of PH in LMICs. This is attributed to financial constraints of their health systems, limited availability of adequate technical infrastructure and equipment, and minimal human expertise in advanced imaging modalities [48]. In fact, in some sub–Saharan African countries, the scenario is dramatic, with limited availability and affordability of essential cardiovascular diagnostic tools, as shown by Jessen et al. [49]. In Mozambique, a low-income country with a population of approximately 30 million inhabitants in 2021, the mean availability of echocardiogram was 33% in the public sector and 66.7% in the private sector in Maputo, the capital city [49].

Cardiac magnetic resonance imaging, single-photon emission computed tomography (SPECT), and positron emission tomography (PET) are well-established non-invasive tests that may add valuable information for the etiological diagnosis and management of PH, particularly due to lung diseases, but are often unavailable in many LMICs.

The work-up of patients with suspected PH should comprise forced spirometry, body plethysmography, lung diffusion capacity for carbon monoxide (DLCO), and analysis of arterial blood gas [9]. However, similarly to other tests, these may be limited in many LMICs—particularly in Africa—due to limited availability of equipment, consumables, and technical support, as well as trained technicians and financial resources [50].

## 6. Proposed Solutions for the Detection and Management of Pulmonary Hypertension in LMICs

### 6.1. Low-Cost Diagnostic Tools

**Figure 3:**
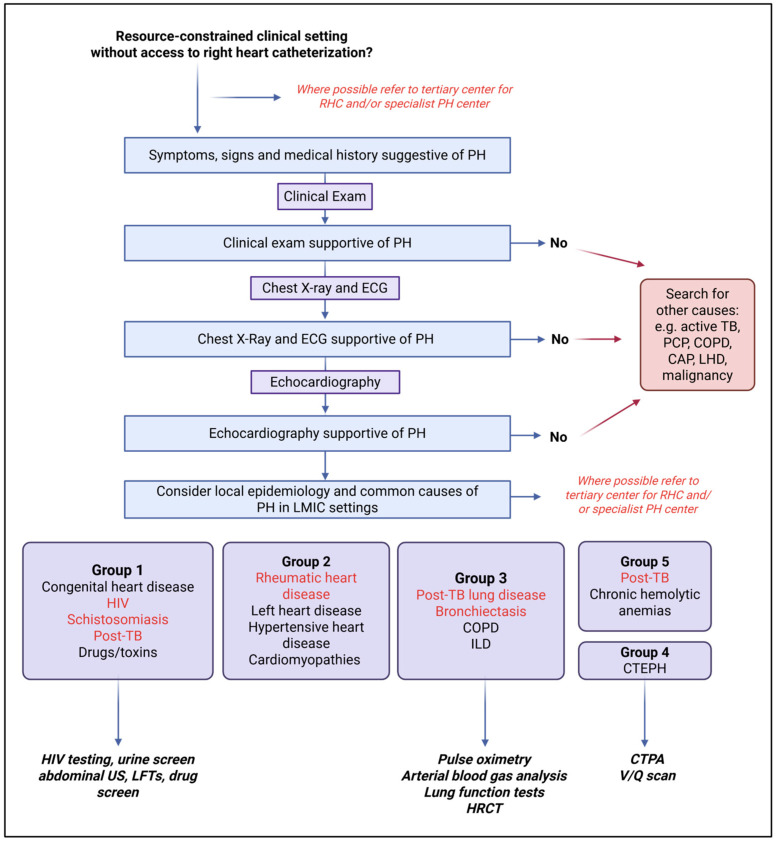
Diagnostic algorithm to diagnose pulmonary hypertension in resource-constrained settings. Adapted with permission. Original figure by Thienemann et al. BMJ Open 2014;4:e005950 [53]. Created with BioRender.com (BioRender, Toronto, Canada, accessed on 19 April 2025). Abbreviations: CAP, Community-Acquired Pneumonia; COPD, Chronic Obstructive Pulmonary Disease; CTPA, Computed Tomography Pulmonary Angiography; HIV, Human Immunodeficiency Virus; HRCT, High-Resolution Computed Tomography; ILD, Interstitial Lung Disease; LHD, left heart disease; LFT, Liver Function Test; MTB, *Mycobacterium tuberculosis*; PCP, Pneumocystis pneumonia; PH, pulmonary hypertension; TB, Tuberculosis; US, Ultrasound; V/Q, Ventilation/Perfusion scan.

Screening algorithms and diagnostic tools used for the detection and diagnosis of PH in low-income settings often need to be modified due to resource limitations. Hasan et al. [42] offer a practical diagnostic algorithm for detection of suspected PH in LMICs without access to cardiac catheterization, focusing on low-cost investigations. If initial history and physical examination, ECG, and chest radiograph are suggestive of PH, echocardiography should be performed and, thereafter, investigation of the etiology, specifically focusing on common causes [44]. We support this approach and propose the algorithm depicted in Figure 3. We emphasize that, where possible, referral to a center with RHC for accurate assessment and diagnosis remains the preferred pathway of care.

For PH in LMICs, we recommend considering infectious disease-associated PH, taking the epidemiology of HIV, schistosomiasis, RHD, and TB into account. For example, HIV rapid testing and/or HIV serology are an essential part of the etiological work-up for those presenting with Group 1 PH in HIV-endemic areas. In addition, liver function tests, urine analysis and abdominal ultrasound are readily accessible tests, which may provide evidence of Sch-PAH as the underlying etiological cause in endemic areas. Similarly, transcutaneous pulse oximetry and arterial blood gas analysis are often available and can provide information regarding parenchymal lung disease, including post-TB lung disease, in those presenting with Group 3 PH. Importantly, in these settings, other common and acute pathologies (including active TB) should be considered and actively excluded. Cardiac catheterization for the diagnosis and/or follow-up of PH remains the gold standard of care, and it is critically important that policy makers and governments prioritize its availability and use in LMICs. In addition, training initiatives should be set up to adequately train medical specialists to perform RHC in these countries. Importantly, RHC should only be performed in specialist centers and by those who are adequately trained, as a lack of expertise and standardization may lead to erroneous data and/or management [44].

Artificial intelligence (AI)—encompassing machine learning (ML) and deep learning (DL)—represents a novel, exciting, and potentially low-cost tool which may transform PH diagnostics and risk stratification. Specifically, AI may allow for more accurate assessments of one or more non-invasive imaging modalities and therefore accurately detect PH without the need for invasive testing, potentially reducing the need for specialist staff and facilities [51]. For example, Liao et al. evaluated the use of ML in echocardiographic assessment of PH and found that their ML model outperformed traditional echocardiographic assessments, with an area under the receiver operator curve (ROC AUC) of 0.945 compared to 0.892 (*p*  =  0.027), respectively [52]. However, for AI models to be reliable in LMICs, researchers must prioritize the creation and utilization of local, high-quality datasets, since AI models trained primarily on data from high-income settings may not generalize accurately to the unique contexts and characteristics of PH in LMICs.

### 6.2. Training and Capacity Building

Healthcare professionals working in primary and secondary hospitals should receive PH-specific training and locally adapted algorithms to facilitate screening and referral to tertiary hospitals. A focal point dedicated to point-of-care echocardiography in primary and secondary care would be important for the screening of PH. Although PH centers in LMICs are scarce, there is a need to increase the number of specialists in PH as well as establish multidisciplinary teams dedicated to improving the management of the disease. More precise data registries from hospitals in LMICs could provide valuable information about the disease, which would allow clinicians to better understand and define priorities to improve the management of the patients. This will also assist the PH community in appreciating the spectrum of disease in vulnerable parts of the world and to develop locally adapted strategies.

## 7. Research Gaps, Remaining Questions and Future Directions

Infectious disease-associated PH remains an under-researched area in PH-specific literature, and thus important research gaps exist. These range from establishing the precise epidemiology and determining how to accurately risk-stratify clinical presentations suggestive of PH to improving outcomes in LMICs. When characterizing these aspects of PH in low-resource settings one needs to consider: can we define and measure PH by the same parameters as those used in higher-resource settings?

We have highlighted some of the potential barriers to the detection of PH in LMICs. However, an acknowledged limitation of this review is its narrative nature with the potential for subjectivity, and more systematic reviews—considering individual etiology-specific and/or region-specific information—are also needed.

In addition, although screening algorithms for PH in LMICs are essential for research purposes, their clinical utility may be limited if access, availability, affordability, and effectiveness of treatments for PH are not ensured. The WHO criteria for the implementation of a screening program specify that “there should be an effective intervention for patients identified through screening” and “there should be agreed evidence-based policies covering which individuals should be offered interventions and the appropriate intervention to be offered”. Therefore, although screening in a clinical context allows data for advocacy and health budgeting and should be considered, implementation of formalized screening programs may be limited by the lack of available treatment for PH in LMICs. Consequently, crucial to the future outcomes of PH in these settings is the development of low-cost therapeutic options. Thus, while novel PH therapies focusing on gene therapy, biologics, and precision medicine are exciting and important in higher-income settings, researchers in this area should also consider more feasible options for use in lower-resourced circumstances.

Bridging the gap between PH-specific care in low- and high-income settings requires collaborative efforts within the PH community. This includes partnerships between high- and low-income settings, knowledge sharing and, importantly, capacity building in LMICs.

## 8. Conclusions

PH is a progressive and potentially life-shortening disease. PH is more common in LMICs compared to high-income settings, with this high burden of disease largely driven by infectious etiologies. Despite its higher prevalence, PH is often underdiagnosed and inadequately treated in lower-income settings due to technical difficulties and limitations associated with detection, diagnosis, and management of PH. This also results in poor outcomes in these settings. It is essential to prioritize low-cost and sustainable solutions for PH in LMICs. Meaningful solutions require innovative changes and collaborative efforts, and will require capacity building and training in LMICs.

## Data Availability

No new data were created or analyzed in this manuscript.

## Abbreviations

The following abbreviations are used in this manuscript:

AI	Artificial intelligence
CTEPH	Chronic thromboembolic pulmonary hypertension
DL	Deep learning
HIC	High-income country
HIV	Human immunodeficiency virus
LMIC	Low- and middle-income country
ML	Machine learning
mPAP	Mean pulmonary arterial pressure
PAH	Pulmonary arterial hypertension
PAPUCO	Pan African Pulmonary Hypertension Cohort study
PASP	Pulmonary artery systolic pressure
PAWP	Pulmonary artery wedge pressure
PH	Pulmonary hypertension
PROKERALA	Pulmonary Hypertension Registry of Kerala
PTLD	Post-TB lung disease
PVR	Pulmonary vascular resistance
RHC	Right heart catheterization
RHD	Rheumatic heart disease
RVOT-AT	Right ventricular outflow tract acceleration time
TB	Tuberculosis
TRV	Tricuspid regurgitant velocity
WU	Wood units
